# Growth of the human eye lens

**Published:** 2007-02-23

**Authors:** Robert C. Augusteyn

**Affiliations:** 1 Institute for Eye Research, Sydney, Australia; 2 Ophthalmology Department, University of Melbourne, Melbourne, Australia

## Abstract

**Purpose:**

To analyze human lens growth from the accumulation of wet weight as a function of age.

**Methods:**

Wet weights were assembled for over 1,100 human lenses, ranging in age from 6 months prenatal to 99 years postnatal, and were examined using various growth models. Initially, prenatal and postnatal data were examined separately, to determine the growth modes and then all data were fitted to a single equation.

**Results:**

Variations in weights due to tissue handling procedures and the unavailability of statistical data for averaged sets precluded the use of >500 values in the present analysis. Regression of age on log lens weight for the remaining 614 lenses indicated that, unlike other species, human lens growth appears to take place in two distinct phases. It was found that asymptotic growth during prenatal life and early childhood generates about 149 mg of tissue in a process, which can be modelled with a Gompertz function. Soon after birth, growth becomes linear, dropping to 1.38 mg/year, and this rate is maintained throughout the rest of life. The relationship of lens wet weight with age over the whole of the lifespan could best be described with the expression, W=1.38A_b_ + 149exp^[exp^(1.6-3A_c_)], where W is lens weight in mg, A_b_ is postnatal age in years and A_c_ is the time since conception in years. Comparison of 138 male and 64 female lenses indicated that there was no statistically significant difference between male and female lens weights in the linear (adult) growth mode.

**Conclusions:**

Human lens growth differs from growth in other species in that it occurs in two distinct modes. The first follows a sigmoidal relationship and provides an initial burst of rapid growth during prenatal development with an apparent termination at or shortly after birth. The second growth mode is linear, adding 1.38 mg/year to lens wet weight, throughout life. Because of the variability in available lens wet weight data, further studies, preferably using lens dry weights or protein contents, will be required to establish precisely when the transition from one growth mode to the other occurs. In contrast to previous reports, it was concluded that, like other species, there are no gender differences in human lens weights.

## Introduction

A thorough comprehension of the biochemical, biometric, optical and physical properties of the human lens, and how these change with age, is essential for understanding the functioning of the eye and the development of age-related visual disorders, such as presbyopia. Many of the required data can only be obtained in vitro, using lenses obtained from eye bank eyes. However, such eyes have generally been stored for several days and their lenses may have become swollen during this time [1]. Because of the difficulty in obtaining fresh human lenses, attempts are sometimes made to extrapolate from animal studies. It is not known whether such extrapolations are always appropriate for modelling human lens properties.

Vertebrate eye lens growth occurs through a unique and ubiquitous mechanism (reviewed in [2]). New epithelial cells, produced just inside the capsule in the equatorial region elongate up to several hundred times during the process of differentiation into fiber cells. Major changes occur in the protein synthesis patterns during this process, notably, the first appearance of β- and γ-crystallins and the ensuing production of large amounts of all three crystallins. The new cells are laid down over existing fiber cells, which are displaced towards the center of the lens. Cellular organelles are lost during maturation of the fiber cells and, concomitantly, most metabolic activity ceases. These processes continue throughout life so that the lens continues to grow larger. In most species, as the mature fiber cells pack into the nuclear region, they become compressed, losing water so that the concentration of protein and, hence, the refractive index increases. Since no cells or their contents (other than water and organelles) are lost, the lens retains a record of its growth and its properties continually change.

Although the same growth mechanism appears to be used in all vertebrate species, there are subtle differences in the rates of growth and in the rates of fiber cell compression. There are also differences in the shape of the lens and in the arrangement of the fiber cells and sutures [3]. As a result, lenses with different properties, appropriate for the specific visual requirements of an animal, are generated. These can range from the very soft avian and reptile lenses, with low refractive index, to the rocklike structures, with very high refractive index, found in rodents and fish [4].

In order to understand the factors which help determine the final properties of a lens, the author has been collecting data on the accumulation of wet weight in the human lens. As can be seen from our previous studies on the kangaroo [5], characterizing the growth pattern requires large numbers of lenses covering the whole age range from foetal to adult life. Because of the difficulties in obtaining human tissues, data collected by the author have been combined with those previously published by a number of laboratories. This communication presents an analysis of these data and conclude that human lens growth differs from growth in other species.

## Methods

Wet weights were obtained in the author's laboratory for over 100 human lenses. Data for >900 more were obtained from the published studies of Bours and Fodisch [6], Broekhuyse [7], Clapp [8], Glasser and Campbell [9], Harding et al. [10], van Heyningen [11], Mach [12], Nordman et al. [13], Pau [14], Rosen et al. [15], Satoh [16], Scammon and Hesdorffer [17], Siebinga et al. [18], Smith [19], and von Jaeger [20]. Unpublished data were provided by Drs J. Harding, B. Ortwerth, J-M Parel, R. Truscott, B. Willekens and G. Vrensen. All of the lenses had been stored under a variety of conditions, some left in the eye for various times, others frozen.

Graphs obtained from the literature were magnified at least ten-fold and the coordinates for the points were measure to the nearest 0.1 mm. The maximum error associated with these measurements was estimated to be 1% for young lenses and less for the older. The relationship between lens weight and age was explored using the linear form of the logistic equation (log Weight=Wm+ b/Age), the Gompertz equation

Weight=Wm*exp⁡^[−exp⁡^(c−dA)]

and by regression of lens weight on the logarithm of age. Wm is the maximum asymptotic weight; A is the age since conception; b, c, and d are constants. The lines of best fit were determined using linear regression.

## Results

**Figure 1:**
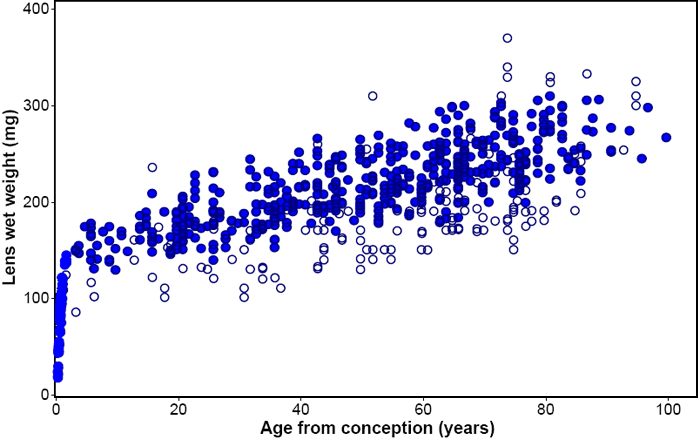
Growth of the human lens. Human lens wet weight plotted as a function of age since conception. Data shown represent >1,100 lenses (open circle), obtained in the author's laboratory and from the literature. The 614 points retained for the present analyses are shown as filled circles.

Figure 1 presents all of the data that the author was able to gather for human lens wet weights. Some of the points are averages so that the 764 points shown represent >1,100 lenses. It is obvious that there are very large variations in lenses of the same age, often as high as ±50% of the mean.

Not all of these published data could be used for the intended analysis of growth. The 104 points from the study by Sieblinga, et al. [18] had to be eliminated because the lenses had been fixed before weighing (G. Vrensen, personal communication). These accounted for most of the lenses with low weights relative to the mean. The 19 averages (207 lenses) from the study by Harding et al. [10] and the 11 averages (239 lenses) described by Scammon and Hesdorffer [17] could not be included in any statistical analysis because there is no truly valid procedure for combining summary data sets, consisting only of means with standard deviations and sample size, with sets of individual data points. In addition, to simplify the analyses, another 20 points, which differed by more than 30% (>4SD) from the mean for that age, were eliminated. Most of these were on the high side of the mean, consistent with lens swelling during eye bank storage.

The remaining 614 lens weights, which were used in the present analyses, are shown as solid circles in Figure 1. The standard deviation for these was around 7% of the mean at each age.

**Figure 2:**
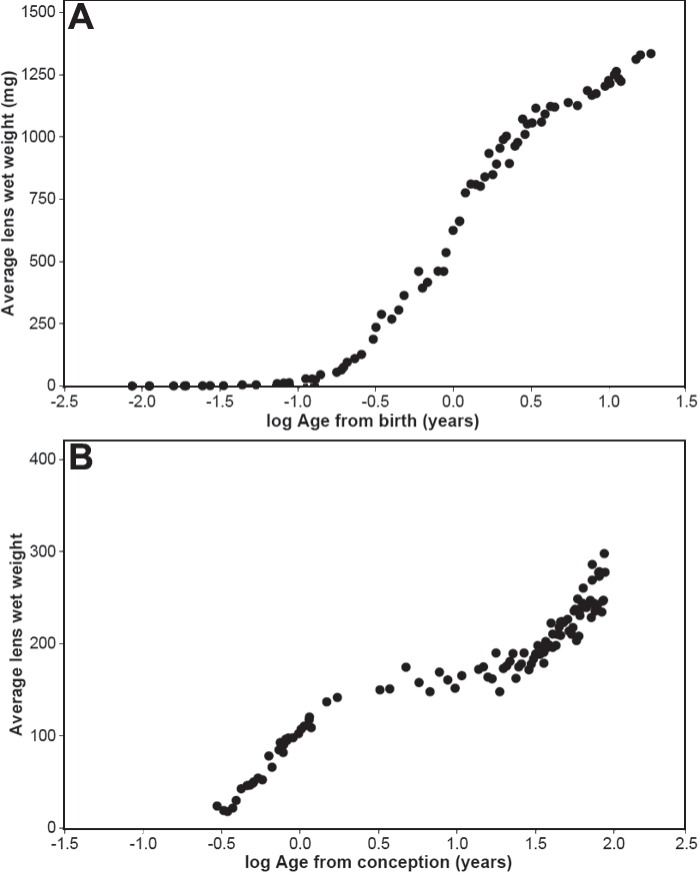
Determination of growth mode. Regression of averaged lens wet weights on log Age for kangaroo (**A**) and human (**B**) lenses.

These data were subjected to curve fitting analysis, using relationships, which have been found satisfactory for many other processes which exhibit self-limiting growth. These included regression of wet weight on log Age and log Wet wt on 1/Age (logistic equation). However, it was immediately clear that, the relationship between age and wet weight was not self-limiting and changed during the life span. This may be seen in Figure 2, which presents the regression of the average lens weights on the logarithm of age since conception (A_c_) and compares this with a similar analysis of kangaroo lenses. Figure 2A demonstrates that the curve for the kangaroo lens is sigmoidal over the whole of the lifespan, approaching a maximum of 1,350-1,400 mg. It would appear that the curve for the human lens is also sigmoidal during gestation and early postnatal life, with an asymptote near 150 mg reached 1-2 years after conception (Figure 2B). However, thereafter, the shape of the curve changes to what may be an exponential. The latter is what would be expected when plotting the logarithm of a linear function.

**Figure 3:**
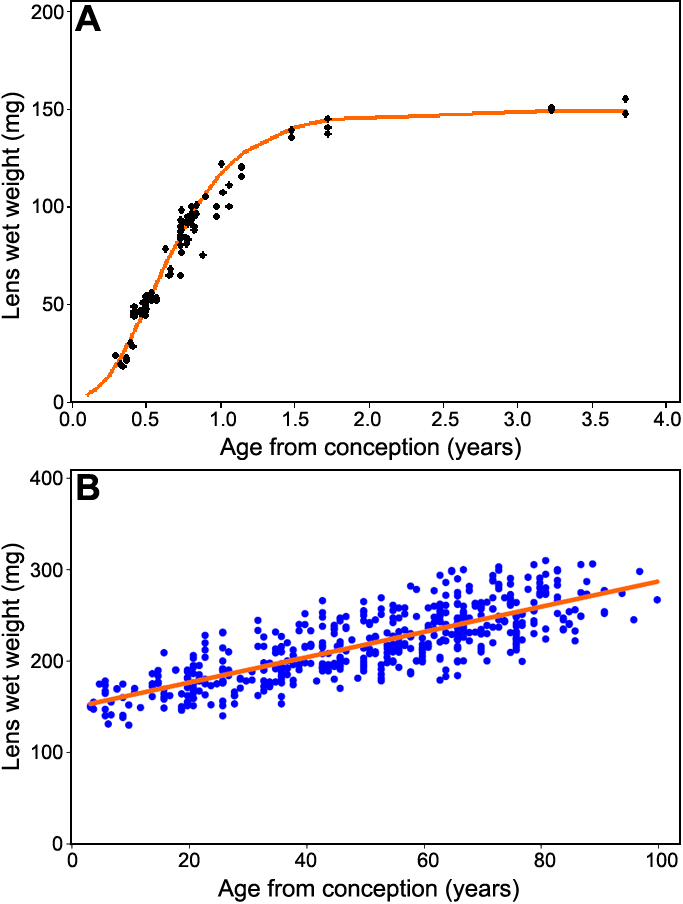
Modelling pre- and postnatal human lens growth. Best fits of the data for (**A**) 96 prenatal and early postnatal lenses using the Gompertz relationship (Wet weight = 149exp^[-exp^(1.6-3A_c_)]; R^2^=0.96) and (**B**) 523 postnatal lenses, aged over 3 years, using a linear relationship (Wet weight=1.38A_b_ + 149; R^2^=0.96).

Because of the apparent change in the growth mode, data from prenatal and postnatal lenses were examined separately (Figure 3). It may be seen in Figure 3A that lens growth is rapid during gestation, generating over 100 mg in the 8 months from lens induction to birth, before quickly slowing down. An apparent maximum of 149 mg is reached at about 1.5 years from conception. This phase of human lens growth can best be described with a Gompertz relationship (R^2^=0.96):

Weight=149*exp⁡^[−exp⁡(1.6−3AC)]

The line of best fit is shown in Figure 3A. The prenatal data, alone, could also be described with a linear fit (R^2^=0.89).

From around age 3 onwards, growth appears to be linear (R^2^=0.64; Figure 3B) and can be described with:

W=1.38A_b_ + 149

A_b_ is the age since birth. Linear growth in adult life has been described previously by Scammon and Hesdorffer [17], Broekhuyse [7] and Harding, et al. [10]. The line of best fit found in the present study is similar to those reported previously.

**Figure 4:**
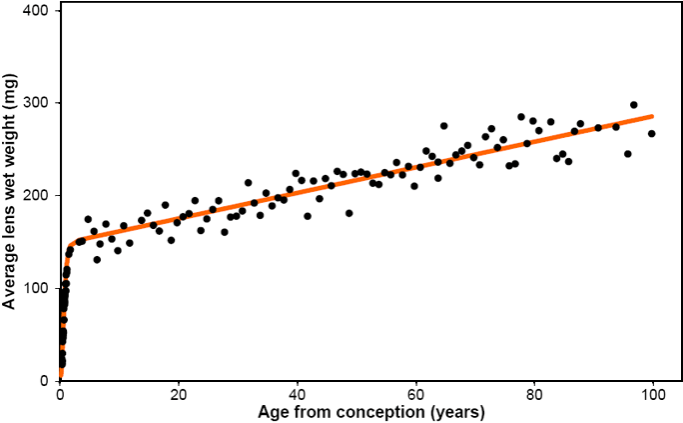
Modelling total human lens growth. Human lens wet weights, averaged for each age, with the best fit of all data according to the equation, Wet weight=1.38A_b_ + 149exp^[-exp^(1.6-3A_c_)].

Combining the two equations provided a description of lens growth throughout life. This is shown in Figure 4.

**Figure 5:**
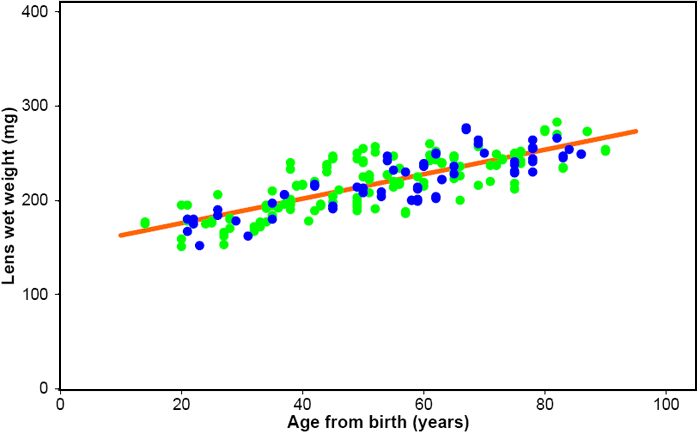
Gender and lens growth. Human lens wet weights for 138 adult male (green filled circles) and 64 adult female (blue filled circles) lenses. Best fits of the data were obtained with Wet weight=1.35A_b_ + 145 (R^2^=0.73) for males and 1.34A_b_ +146 (R^2^=0.72) for females. The line of best fit obtained with all postnatal lenses (Wet weight=1.38A_b_ + 149; Figure 3B) is included for comparison.

The data assembled for this study included weights for 64 female and 138 male adult lenses. These have been plotted in Figure 5 and may be compared with the linear component of the growth curve for all adult lenses (Figure 3B), which has been included. The data seem to be indistinguishable but the linear fits [male lens wt=1.34A_b_ + 149 (R^2^=0.73); female lens wt=1.33A_b_ + 145 (R^2^=0.71)] suggest that there might be a very slight difference between the two. However, the difference was not significant for either slope (p=0.46) or intercept (p=0.51).

## Discussion

Although the data collected were derived from over 1,100 human lenses, nearly half had to be eliminated. Most of these were data only available as averages with insufficient statistical information to permit their correct reanalysis. Lenses which had been fixed in formalin before weighing were also eliminated. Recent experiments (Willekins, Vrensen and Augusteyn, in preparation) have shown that formalin fixation results in substantial water losses, up to 21% in 7 days. In addition, lenses which were substantially heavier (>4 SD above the mean) were judged to be severely swollen and were left out. Even after elimination of the above data, there were still substantial variations. This variability can, in part, be attributed to cataractous changes which can result in both increases and decreases in lens weight, but, most likely, it is due to post-mortem uptake of water. We have shown that sheep lenses left in the eye in the cold, rapidly accumulate water [21], as do human lenses left in the eye in the eye bank [1]. As noted previously, this accumulation of water will compromise the lens and may invalidate observations made on these lenses. It is likely that the estimates of lens growth rates, in the present study, will be high because of such swelling. This is suggested by the analysis of the known male and female lenses, which all showed a lower rate of wet weight accumulation (1.34 mg/year compared with 1.38 for all lenses). Most of these came from the study by Smith [19]. It is probable that these lenses were examined at a shorter time, post mortem, than more recent samples.

The analyses presented here, indicate that human lens growth takes place in two distinct phases; asymptotic during gestation and early childhood, followed by a linear increase thereafter. This is different from all other species examined to date, in which lens growth is asymptotic throughout the whole of the life span. Such growth can be described with a simple logistic relationship whereas human lens growth requires a combination of Gompertz and linear functions.

A two-phase growth pattern may also be inferred from the conclusions of Scammon and Hesdorffer [17] who noted that the lens showed "a curious pattern of growth". They observed that, "In infancy and early childhood, the growth follows the pattern common to most parts of both the central and peripheral nervous system. But thereafter growth is continued at a steady rate, whereas most structures associated with the nervous system approach or reach their definitive size in the second decade."

The prenatal data could also be adequately described with a linear relationship. However, this may reflects the lack of data from early embryonic stages. Embryonic growth is not a linear process and lens growth does not commence until Carnegie stage 13, around 28 days after conception, with the formation of the lens placode. However, it is not until around stage 22 (54 days) that a cellular lens is evident and lens weight can start to increase. Intuitively, one would expect this to be a gradually-accelerating process. In addition, it should be remembered that compression of lens cells, with subsequent loss of water, may be taking place so that the measured wet weight is not indicative of the amount of tissue originally laid down. Further information, especially on the increase in dry weight, is needed to assess the possibility of prenatal linear growth. Current information favours the asymptotic model.

The asymptote maximum is reached at 6-9 months after birth. Thus, the growth rate changes abruptly from an average rate of >150 mg/yr, late in gestation, to 1.38 mg/yr, a 100 fold decrease over a period of less than one year. The signal for this is most likely to be associated with birth since there would be a delay before its effects would become obvious. Cells generated late in gestation would continue to divide and differentiate at the prenatal rate until they had all formed mature fiber cells. Interestingly, gamma crystallin synthesis in the human lens ceases immediately after birth, unlike other species where it continues to be produced postnatally [22]. This suggests that both the rate of cell deposition and the complement of proteins synthesized by the fiber cells are altered at birth.

It is difficult to ascertain why there should be two growth phases. Clearly, the prenatal growth rate could not be sustained in postnatal life. However, in other species, the transition is a more gradual one and lens sizes asymptote to finite values in adult life. It may be that the two growth modes are required to generate regions with different properties within the lens. The central region of the adult human lens contains a distinct nucleus, measuring around 3 by 7 mm [23] and bounded by a diffusion barrier [24]. It is formed by a 2-3 fold compression of older fiber cells and has a constant refractive index [4]. This nucleus is substantially stiffer than the surrounding tissue [25], possibly related to its γ-crystallin content. By contrast, the outer, cortical region of the lens is relatively soft, lacking γ-crystallin, and has a gradient of refractive index. It has no finite boundaries, continuing to grow throughout life by accumulating 1.38 mg wet weight per year. This results in small but significant increases in both sagittal thickness and equatorial diameter [1]. What the functional outcome of this arrangement may be remains to be determined in the context of growth in other ocular tissues.

It is tempting to speculate that the nucleus represents those fiber cells laid down during the period of rapid growth and that the diffusion barrier reflects the change in cell properties following the transition to linear growth. Unfortunately, the variability in the available data does not permit determination of the precise time of the transition. In addition, because the nucleus is formed by compression, the adult wet weight and volume will be considerably lower than they were when the tissue was laid down or even at the time of the transition. However, since there is no turnover of protein in the lens, the protein content or dry weight offer a more reliable measure of tissue age which may resolve these questions. Such data are currently being accumulated by the author and associates.

It has been reported that men have larger lenses than women [10]. It has also been reported that male and female beagle dogs [26] fur seals [27], grouse [28], pheasants [29] and Han-Wistar rats [30], have different sized lenses. However, re-examination of most of these data does not reveal convincing differences. In addition, several other studies on the rat, as well as studies on a variety of species (bandicoot, bat, carp, deer, dingo, dog, dogfish, dunnart, elephant, lemming, mountain hare, kangaroo, mouse, possum, rabbit, and sheep) have indicated that there are no gender differences in lens size. The current analyses, as well as our preliminary observations on rhesus monkeys and baboons, indicate that primate lens sizes are also the same in males and females. In view of these various observations, it is concluded that there are no gender difference in lens size in humans or in other species.

Much of the data, collected in the present study, came from published works describing biochemical, biometric, physical and/or optical properties of isolated human lenses. Many of these studies continue to be cited. The variability in lens weight noted in the present study indicates that the properties of many of these lenses may not have been indicative of their in vivo condition. At least 20% of lenses from eye bank eyes have high weights due to swelling during eye bank storage [1]. This is an inescapable consequence of working with human tissues. As pointed out previously, caution needs to be exercised when interpreting data from such lenses.
